# An Interventional Study on the Late Treatment of Severe Bronchopulmonary Dysplasia in Preterm Infants Using Mesenchymal Stromal Cells

**DOI:** 10.1155/sci/2715294

**Published:** 2026-01-08

**Authors:** Sukran Yildirim

**Affiliations:** ^1^ Neonatology Clinic, University of Health Sciences, Prof. Dr. Cemil Tascioglu City Hospital, Istanbul, Türkiye, akdeniz.edu.tr

**Keywords:** BPD, late treatment, mesencymal stromal cell, prematurity

## Abstract

**Background:**

Bronchopulmonary dysplasia (BPD) remains a significant challenge in the management of preterm infants. Although conventional treatment approaches have helped reduce the morbidity associated with BPD, the prevalence of the condition has not decreased, highlighting an urgent need for new therapies. Mesenchymal stromal cell (MSC) therapy has emerged as a potentially promising intervention, showing a favorable safety profile in Phase II clinical trials. However, research on the use of MSCs as a late‐term therapeutic strategy for established BPD is still in its early stages, indicating a need for further studies to evaluate their effectiveness and optimal application.

**Aim:**

To investigate the results of seven extremely preterm infants who underwent an MSC therapy for severe established BPD.

**Method:**

A cohort of seven infants diagnosed with severe BPD (sBPD) received MSC therapy to assist in their transition to spontaneous breathing. For the first six infants, MSC therapy was discontinued after extubation; however, the final infant continued to receive MSC therapy as he remained on nasal continuous positive airway pressure (nCPAP).

Each treatment cycle involved administering 1 million MSCs per kilogram via intratracheal injection, along with an additional 0.5 million MSCs delivered through intravenous infusion. Treatment was initiated between postnatal days 32 and 84. The first three infants each underwent two treatment cycles, the fourth infant received three cycles, and the last three infants were scheduled for 4 weekly cycles. A retrospective analysis was conducted to evaluate the outcomes of the therapy.

**Results:**

All infants were successfully extubated to nCPAP following MSC treatment. However, two infants who underwent two cycles of MSC therapy could not be weaned off respiratory support. In contrast, all infants who received three to four cycles were successfully weaned off the ventilators and discharged home without the need for supplemental oxygen. Additionally, secondary benefits were observed, including improvements in intraventricular hemorrhage (IVH) and retinopathy of prematurity (ROP), a decrease in the number of packed red blood cell transfusions, and fewer episodes of sepsis.

**Conclusions:**

Our findings indicate that administering up to four treatment cycles may be more effective for the long‐term management of sBPD. Additionally, using MSCs through both intratracheal and intravenous routes could offer benefits beyond just the lungs, highlighting an area for further research.

## 1. Introduction

Mesenchymal stromal cells (MSCs) play a crucial role in regenerative medicine due to their ability to replicate, differentiate, and migrate to injured tissues, making them ideal candidates for tissue regeneration [1]. Recent research has shown that the primary regenerative effects of MSCs are mediated through their paracrine activity, which involves the release of extracellular vesicles [2]. This insight, along with concerns about potential long‐term issues such as tumorigenicity, has led to an increasing interest in exosomal therapies, also known as acellular therapies [3]. However, cellular therapies have a significant advantage, as evidenced by the long‐lasting effects observed in animal studies [4, 5]. There is still much to learn about the use of cellular and acellular therapies involving MSCs [1, 3].

In the field of neonatology, human umbilical cord‐derived mesenchymal stem cells (HUC‐MSCs) are emerging as a promising treatment for bronchopulmonary dysplasia (BPD) [6–18], a serious condition commonly associated with premature birth [19]. BPD is often linked to several severe comorbidities, including intraventricular hemorrhage (IVH), necrotizing enterocolitis (NEC), retinopathy of prematurity (ROP), the need for multiple blood transfusions, and sepsis [20, 21]. Each of these conditions independently increases the risk of adverse long‐term neurodevelopmental outcomes [22–28]. MSCs have several beneficial properties, including pro‐angiogenic, anti‐fibrotic, anti‐inflammatory, antiapoptotic, antimicrobial, and immunomodulatory effects [29, 30]. These characteristics hold potential for addressing various disorders related to prematurity, including BPD and its associated comorbidities [31].

Research by Kim et al. [32] has demonstrated that the intratracheal transplantation of MSCs can reduce brain injury in rats while enhancing lung function. Additionally, a study indicates that preterm infants who receive intravenous MSC therapy for BPD experience improved immune function [15]. The encouraging results from these studies suggest that the benefits of MSC therapies may extend beyond their initial targets [15, 32].

In our study, we performed a thorough analysis of clinical outcomes for seven extremely preterm infants who received late‐term MSC therapy for severe BPD (sBPD). We hypothesized that MSC treatment would facilitate extubation and have a positive impact on other related conditions in these infants.

## 2. Materials and Methods

We analyzed the data of seven preterm infants born at Prof. Dr. Cemil Tascioglu City Hospital and admitted to our Neonatal Intensive Care Unit between 2020 and 2024, all of whom received allogeneic HUC‐MSC treatment for sBPD (Tables 1–3).

**Table 1:** Baseline demographic data of the infants.

Data	Baby 1	Baby 2	Baby 3	Baby 4	Baby 5	Baby 6	Baby 7
Gender	Female	Male	Male	Male	Female	Female	Male
Gestational age at birth (weeks)	28	24 6/7	26 2/7	27 6/7	25 2/7	23 6/7	24 3/7

Birthweight (g) (percentile)	800 (18 p)	652 (28 p)	1070 (90 p)	1131 (63 p)	511 (6 p)	625 (62 p)	725 (62 p)

Prenatal/ natal history	IVF Preeclampsia gestational hypertension, PPROM, oligohydramnios, chorioamnionitis, and emergency C/S (uncontrolled maternal hypertension)	PPROM, premature labor, and emergency C/S (fetal distress)	PPROM, oligohydramnios, urinary tract infection in the last trimester, premature labor, and emergency C/S (fetal distress)	Gestational hypertension, gestational diabetes mellitus, SARS‐CoV‐2 infection and cholecystitis in the past 20 days, and emergency C/S (fetal distress)	Cervical incompetence, normal spontaneous vaginal delivery	Oligohydramnios, chorioamnionitis, and emergency C/S (fetal distress)	Preeclampsia led to intracranial bleeding in the mother, emergency C/S due to the mother’s critical condition

Abbreviations: C/S, cesarean/section; g, gram; IVF, in vitro fertilization; p, percentile; PPROM, preterm premature rupture of membranes.

**Table 2:** Short‐term respiratory outcomes after MSC treatment.

Outcome	Baby 1	Baby 2	Baby 3	Baby 4	Baby 5	Baby 6	Baby 7
Postnatal/postmenstrual age at the start of MSC treatment (days/weeks)	84/40	55/32 5/7	63/35 2/7	49/34 6/7	32/29 6/7	68/33 4/7	64/33 4/7
MSC treatment cycles	2	2	2	3	4	4	4
Time interval between MSC treatment cycles (days)	15	15	15	7	7	7	7
Respiratory status at the beginning of MSC therapy	Invasive ventilation with a FiO2 of 40%	Invasive ventilation with a FiO2 of 40%	Invasive ventilation with a FiO2 of 70	Invasive ventilation with a FiO2 of 40%	Invasive ventilation with a FiO2 of 45%	Invasive ventilation with a FiO2 of 30%	Noninvasive ventilation with a FiO2 of 40%
Postnatal age (day)							
Extubated to noninvasive ventilation	134	84	98	70	89	79	60
Spontaneous breathing with supplemental oxygen	–	104	–	73	103	100	93
Room air	–	107	–	74	146	140	126

Abbreviation: MSC, mesenchymal stromal cell.

**Table 3:** Secondary out comes for infants before and after MSC treatment.

Outcome	Baby 1	Baby 2	Baby 3	Baby 4	Baby 5	Baby 6	Baby 7
Echocardiography							
Before MSC treatment	Pulmonary hypertension, (the pulmonary artery pressure: 50 mmHg^1^)	Pulmonary hypertension (the pulmonary artery pressure: 45 mmHg^1^)	Pulmonary hypertension, (the pulmonary artery pressure: 40 mmHg^1^)	NA	NA	NA	NA
After MSC treatment	Pulmonary hypertension (the pulmonary artery pressure: 35 mmHg^1^), 30% decrease 1 week after MSC therapy	Pulmonary hypertension (the pulmonary artery pressure: 30 mmHg^1^), 33% decrease one 1 after MSC therapy	Pulmonary hypertension, (the pulmonary artery pressure: 30 mmHg^1^), 25% decrease 1 week after MSC therapy	NA	NA	NA	NA
The total number of PRC transfusions (mean number per month)							
Before MSC treatment	7 (2,5)	9 (4,9)	8 (3,8)	6 (3,7)	8 (7,5)	14 (6,2)	11 (5,2)
After MSC treatment	4 (0,8)	3 (1,3)	7 (0,8)	1 (1)	6 (1,6)	2 (0,7)	1 (0,3)
The total number of courses of antibiotics (mean number per month)							
Before MSC treatment	6 (2,1)	5 (2,7)	4 (1,9)	4 (2,4)	2 (1,9)	5 (2,2)	2 (0,9)
After MSC treatment	8 (1,6)	1 (0,4)	12 (1,3)	0	3 (0,8)	0	0
Retinopathy of prematurity							
Before MSC treatment	Zone II stage III	Zone I stage 0	Zone II stage II, preplus(+)	Zone II stage II	Zone I stage 0	Zone II stage III, preplus (+)	Right eye zone I, stage 0 left eye zone II, stage II, plus (+)
After MSC treatment	Zone III stage I on postnatal day 108	Zone III stage I on postnatal day 79	Zone II stage II, no pre plus on postnatal day 70	Zone II stage I, on postnatal day 59	Zone II stage II, pre plus (+), anti VGF injection (once) Zone II stage I at the time of discharge	Zone II, Stage III, pre plus (+) laser treatment (twice) on postnatal day 137 and 142	Zone II Stage II, no plus at postnatal day 90
Cranial ultrasound findings							
Before MSC treatment	No IVH	No IVH	IVH grade I, ventriculomegaly (levene index 0,36)	IVH grade II	IVH grade I Mild PVL on postnatal day 26	No IVH	IVH grade III
After MSC treatment	Normal	Normal	Ventriculomegaly (levene index 0,45)	Normal	Normal	Normal	Normal

*Note:* Pulmonary arterial pressure was measured from the systolic measures from tricuspit regurgitation.

Abbreviations: IVH, intraventricular hemorrhage; mmHg, millimeter of Mercury; MSC, mesenchymal stromal cell; NA, not applicable; PRC, packed red cells.

The study’s inclusion criteria were

1. Infants born at or before 28 weeks of gestation.
2. Infants needing ventilatory support at 1 month of age.
3. Exhaustion of all conventional therapies.

Exclusion criteria included:

1. Major congenital malformation.
2. Lack of parental consent or a grant from the Turkish Ministry of Health.
3. Not meeting eligibility.

**Figure 1:**
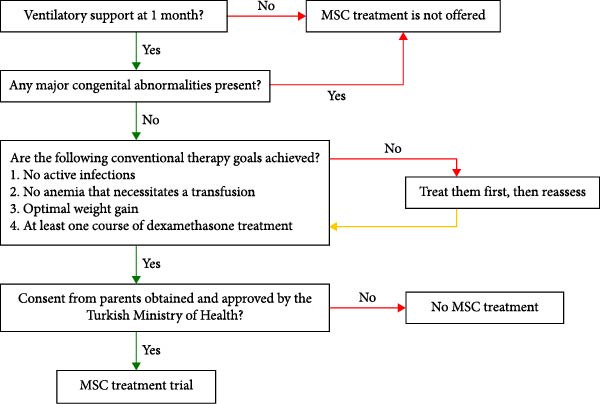
Eligibility criteria for infants receiving MSC treatment.

Parents were informed of the criteria and offered HUC‐MSC treatment after their baby reached 1 month of age and had been confirmed eligible. The eligibility criteria are outlined in Figure 1.

The MSCs were manufactured under current good manufacturing practice conditions by Acibadem Labcell, an accredited and certified laboratory functioning as a subdivision of Acibadem Labmed, a unit of the Acibadem Healthcare Group (Istanbul, Turkey). The release criteria for MSC products are outlined in Supporting Information 1. Approval has been granted by the Turkish Ministry of Health. The approval numbers are provided in Supporting Information 2.

Dexamethasone treatment was administered as described by Doyle et al. [33]. The need for packed red blood cell transfusions was evaluated following the Turkish Neonatal Society guidelines [34]. Active infections were assessed based on the European Medicines Agency guidelines [35]. An experienced pediatric cardiologist determined the presence of a PDA. Satisfactory weight gain was defined as a minimum of 15 g/kg per day, as described by Steward [36]. A skilled pediatric neurologist evaluated the infants’ neurodevelopment. Following this assessment, the hospital’s Medical Board for Disabilities confirmed a diagnosis of cerebral palsy. After the diagnosis, the family was offered special education services funded by social insurance.

The administration dosing was structured as follows: Each treatment cycle consisted of delivering 1 × 10^6^ cells/kg of MSCs via intratracheal injection. Additionally, 0.5 × 10^6^ cells/kg of MSCs were administered intravenously through a peripheral vein. The intravenous MSCs were diluted in 30 mL of saline and infused 30 min after the intratracheal injection.

The first three infants received two treatment cycles, each separated by 15 days. The fourth infant underwent three treatment cycles, scheduled 1 week apart. The last three infants participated in four treatment cycles, scheduled 1 week apart (Table 2).

## 3. Results

The infants were born between 23 weeks and 6 days to 28 weeks, with birth weights ranging from 511 to 1131 g. Six of the infants were born via emergency C/S due to either fetal distress or the mother’s critical condition; one infant, whose mother had cervical incompetence, was delivered vaginally. A detailed description of the baseline demographic data is displayed in Table 1.

At the beginning of treatment, six out of seven infants were on invasive ventilation, and one was on noninvasive ventilation, with fractional inspired oxygen (FiO2) levels ranging from 30% to 70%. The goal for the first six patients was to extubate them to noninvasive ventilation, while the aim for the final infant was to improve spontaneous breathing. All infants achieved these objectives; however, Baby 1 could not be weaned off oxygen, and Baby 3 could not be weaned off noninvasive ventilation. The parents of Baby 3 decided not to continue with HUC‐MSC treatment due to financial concerns and were transferred to the pediatric intensive care unit while still on noninvasive ventilation. Baby 1 was discharged with supplemental oxygen. Ultimately, five of the infants were discharged on room air. Short‐term respiratory outcomes from the MSC treatment are summarized in Table 2.

Table 3 elucidates secondary clinical benefits documented in the study, which were as follows:

1. A significant regression of pulmonary hypertension was observed in the first three infants just 1 week post‐initiation of MSC therapy.
2. Every patient demonstrated a diminished necessity for packed red blood cell transfusions alongside a reduced requirement for antibiotic administration after the treatment.
3. Regression of ROP was recorded in Babies 1, 3, 4, and 7.
4. Regression of IVH was noted in Babies 3, 4, 5, and 7.

One preterm infant passed away at 12 months from sepsis, and two others were diagnosed with mild cerebral palsy. Four out of the seven infants exhibited normal neurodevelopment. Long‐term outcomes are detailed in Table 4.

**Table 4:** Long‐term outcomes for infants who received MSC treatment.

Outcomes	Baby 1	Baby 2	Baby 3	Baby 4	Baby 5	Baby 6	Baby 7
Current chronological age	5 years	4 years 10 months	NA	4 years	3,5 years	1 year	1 year
Issues following discharge	Recurrent respiratory infections (2 pediatric intensive care unit admissions)	Growth hormone deficiency	Recurrent respiratory infections	None	None	1 hospitalization for respiratory infection	1 pediatric intensive care unit admission for respiratory infection
Death	No	No	Yes, passed away at the age of 12 months due to sepsis	No	No	No	No
Long‐term neurodevelopment	Mild cerebral palsy	Normal	NA	Mild cerebral palsy	Normal	Normal	Normal

Abbreviation: NA, not applicable.

## 4. Discussion

In medicine, it is widely recognized that prevention is preferable to treatment [37]. Clinical trials exploring MSC therapy for preventing BPD support this notion. While MSCs show promise in aiding the prevention of BPD, only a limited number of Phase II trials have been completed currently [13, 15, 16], and none were completed at the time of the trial of MSC treatment in our first three patients. Consequently, we have decided to offer this new treatment to our patients only after all conventional therapies have been exhausted (Figure 1). As a result, our study focused on using MSCs as a treatment option rather than a preventive measure. This decision led us to administer the therapy later in the disease’s progression. We noted a lack of published data regarding the late administration of MSC treatment for patients with established BPD. Lim et al. [8] administered a dose of 0.5–1 million human amnion epithelial cells (hAECs) per kg to six extreme preterm infants with BPD via intravenous injection when the infants were, on average, 89 days postnatal (with a range of 59–187 days). After the treatment, they did not observe any improvements in respiratory function, and five infants were discharged home with supplemental oxygen. However, the researchers noted microembolic events related to the cells following the manual intravenous administration of hAECs. They switched to a 30‐min infusion method for the subsequent five infants to address this issue. Overall, they concluded that the administration of hAECs was safe. Álvarez–Fuente et al. [9] administered two different doses of intravenous allogeneic bone marrow‐derived MSCs to two infants with sBPD over 85 postnatal days to 5 months. The doses were repeated twice to three times, a week apart. While no respiratory improvement was observed, there was a mild improvement in pulmonary hypertension and a reduction in inflammatory biomarkers in peripheral blood cells. Unfortunately, both patients died despite the therapy [9]. Although the initial results with hAECs and bone marrow‐derived MSCs were disappointing, research continued with HUC‐MSCs. Nguyen et al. [12] administered an intravenous dose of 1 million cells/kg to three extremely premature infants between postnatal days 114 and 160. These infants received oxygen through nasal cannulas at a flow rate of 0.5–1 L/min. After the first dose of HUC‐MSCs, the researchers found that the infants became independent of oxygen within a period ranging from 4 days to 2 months. Additionally, there was a significant reduction in lung fibrosis and improved lung function [12]. Recent studies indicate that MSC therapy is a safe option for treating BPD [6–18], providing hope even for challenging cases in advanced stages [9, 12, 18]. However, there are still insufficient data to establish the optimal timing and the long‐term outcomes of MSC treatment [18]. We administered two cycles of MSCs to the first three infants due to the parents’ financial concerns and the limited information available, particularly regarding the effects of repeated doses from 2020 to 2021. Despite these obstacles, all three patients were successfully extubated and transitioned to nasal continuous positive airway pressure (nCPAP) between 21 and 38 days after the initial MSC therapy cycle. Also, pulmonary artery pressures were reduced within 1 week, similar to the findings of Álvarez–Fuente et al. [9] (Table 2). However, two of the three babies could not be weaned off respiratory support. Only one baby was free of oxygen support 56 days after receiving the first dose (Table 2). In contrast, all the babies who received three to four cycles of MSCs were free from oxygen support between 27 and 106 days after their first dose (Table 2). These findings suggest that giving three or more doses may enhance outcomes for the most severe cases. O’Reilly et al. [5] demonstrated that a single dose of MSC treatment only partially reduced oxygen‐induced lung injury, while multiple doses of MSCs had a more significant effect in animal studies. Similar results were observed in other experimental models of chronic lung disease [38–40].

sBPD is frequently associated with significant comorbidities [20]. Treating BPD with MSCs may offer a promising strategy to address these related health issues simultaneously. We hypothesize that administering MSCs intratracheally before intravenous delivery could saturate the lung capillaries. This approach may enhance the flow of MSCs to other organs, potentially providing additional therapeutic benefits.

We observed a decrease in the need for red blood cell transfusions in all patients after treatment with MSCs (Table 3). One possible explanation for this finding is the restoration of bone marrow function by MSCs, as demonstrated by Carranccio et al. [41] and Zhang et al. [42] in animal models. Additionally, HUC‐MSCs have been used effectively to treat hereditary bone marrow failure syndromes in pediatric patients [43]. Another possible mechanism for the decreased transfusion requirement involves the restoration of erythropoietin production in the liver. During human fetal development, liver macrophages are the primary producers of erythropoietin [44], and MSCs are suggested to influence the basic functions of macrophages [45].

In this study, another secondary outcome was a notable decrease in septic episodes following MSC treatment (Table 3). MSCs have been shown to possess antimicrobial properties and can enhance survival rates and promote bacterial clearance in animal models [46, 47]. Research by Cerro Marín et al. [17] and Zhuxiao et al. [15] demonstrated improvements in specific immune cells and inflammatory biomarkers in both blood and lung tissue after MSC therapy in preterm newborns with BPD. Despite a reduction in septic episodes, most of the babies had recurrent respiratory infections in long‐term follow‐up (Table 4), so the immunomodulatory effect of MSCs seems to be temporary.

Finally, regression of ROP was observed in four patients: 1, 3, 4, and 7. Additionally, resolution of IVH was noted in patients 3, 4, 5, and 7 (Table 3). While animal studies using MSCs for treating ROP have shown promising results, there are limited data on their use in human preterm infants [48]. Furthermore, a Phase I trial investigating the use of MSCs for IVH indicated that a larger, controlled study is necessary [49].

What is already known:

1. HUC‐MSCs are safe for use in newborns, with no adverse effects reported up to a 5‐year follow‐up.
2. HUC‐MSCs may help facilitate extubation in preterm newborns with BPD.

What this study adds:

1. Repetitive doses of HUC‐MSCs may provide additional benefits for patients with established BPD.
2. HUC‐MSC therapy for BPD may have effects beyond the lungs, which have not been previously reported.

While we are encouraged by the positive clinical outcomes observed, we acknowledge that this study has several limitations that must be considered:

1. It is a retrospective and descriptive study conducted at a single center with a small sample size and no control group, making it challenging to establish a causal relationship between MSC treatment and outcomes. Additionally, since there is no defined natural progression of the disease, we cannot determine whether the outcomes would have been the same with or without treatment.
2. While we hypothesized that administering MSCs both intratracheally and intravenously could yield further benefits—evidenced by improved clinical results—we did not investigate the distribution of MSCs to organs beyond the lungs. This investigation would require invasive procedures, which could potentially harm patients and raise ethical concerns.

Further implications:

Randomized controlled trials are essential for assessing the effects of repeated doses of MSC treatment on established BPD, as well as for evaluating secondary outcomes. Additionally, animal studies and histopathological investigations can offer valuable insights into the distribution of MSCs beyond the lungs.

## 5. Conclusion

Our findings support the potential safety of intravenous and intratracheal MSC therapy for BPD in preterm neonates. We suggest that administering repetitive doses may be more beneficial when utilizing MSCs as a late‐term therapy for sBPD. We highlight potential secondary benefits, although the available data on this topic are limited.

## Conflicts of Interest

The authors declare no conflicts of interest.

## Funding

No funding was received for this manuscript.

## Supporting Information

Additional supporting information can be found online in the Supporting Information section.

## Supporting information


**Supporting Information 1** The criteria established by the manufacturer include specifications for label content, viability, cell count, purity analysis, efficacy, cytogenetics, toxicity, stability, and microbiological quality control. These release criteria are based on the specific validation requirements set forth by the manufacturer.

**Supporting Information 2** The approval numbers assigned to each patient by the Turkish Ministry of Health.

## Data Availability

The data that support the findings of this study are available upon request from the corresponding author. The data are not publicly available due to privacy or ethical restrictions.
