# Supplementary material for: An Interventional Study on the Late Treatment of Severe Bronchopulmonary Dysplasia in Preterm Infants Using Mesenchymal Stromal Cells
Source: Stem Cells Int. 2026 Jan 8;2026:2715294. doi: 10.1155/sci/2715294 (PMC12783681; doi:10.1155/sci/2715294)
Supplement: Supplementary file 1 — Supporting Information 1 The criteria established by the manufacturer include specifications for label content, viability, cell count, purity analysis, efficacy, cytogenetics, toxicity, stability, and microbiological quality control. These release criteria are based on the specific validation requirements set forth by the manufacturer. [file SCI-2026-2715294-s001.docx]

| **Supplementary Material 1:** Manufacturer's Release Criteria for Mesenchymal Stromal Cells | | | |
| --- | --- | --- | --- |
| **PARAMETER** | **METHOD** | **LIMIT** | **EXPLANATION/RESULT** |
| Label content | Observe | - | Verify the giver-taker protocol and the patient’s name on the product label. The name of the giver must not appear on the label of products sent to third parties. |
| Viability | Trypan blue staining | > 80% | The upper limit is not important for viability.  5ml vials contain 25x10^6^/cells (±10%),  20 ml vials contain 40x10^6^/cells (±10%). |
| Cell count | Microscopic/CBC | For one cycle of production:  >50x10^6^/cells |  |
| Purity analysis | | | |
| CD34 | Flow Cytometry | <2% | - |
| CD45 |  | <4% | - |
| CD14 |  | ≤2% | - |
| CD19 |  | ≤2% | - |
| CD11b |  | ≤2% | - |
| CD90 |  | >80% | - |
| HLA-DR | Flow Cytometry | <4% | - |
| CD105 |  | >60% | - |
| CD73 |  | >70% | - |
| Effectivity analysis | | | |
| Cytogenetic analysis | Cell culture | Normal | Investigate chromosomal abnormalities in all samples from the fourth passage and one sample from the third passage among five products. |
| Microbiological quality control | | | |
| Microbiological quality control | Culture | Negative | A culture is taken 24 hours before the product is finished and at the time of completion. Observe for 14 days. A sample is collected from the product's supernatant at release, stained with Wright's and Giemsa's stains, and examined under a microscope. |
| Mycoplasma | PCR | Negative | A PCR analysis for Mycoplasma is conducted one day prior to product release, utilizing a sample taken from the culture collected the previous day. |
| Fungal culture | Culture | Negative |  |
| Toxicity analysis | | | |
| Endotoxin analysis | Gel Clot | For IV products  <5/kg or <50 Ü total. For IT products 0.2 U/kg or <10 Ü total. | I.V. :intravenous, and I.T. : intrathecal. |
| Stability Analysis | | | |
| Stability | Trypan Blue | >80% | Viability at 48 hours |
