# Supplementary material for: An Interventional Study on the Late Treatment of Severe Bronchopulmonary Dysplasia in Preterm Infants Using Mesenchymal Stromal Cells
Source: Stem Cells Int. 2026 Jan 8;2026:2715294. doi: 10.1155/sci/2715294 (PMC12783681; doi:10.1155/sci/2715294)
Supplement: Supplementary file 2 — Supporting Information 2 The approval numbers assigned to each patient by the Turkish Ministry of Health. [file SCI-2026-2715294-s002.docx]

**Supplementary Material 2.** Approval numbers from the Turkish Ministry of Health for each patient.

1. 12.01.2021-56733164/203
2. 12.01.2021-56733164/203
3. 26.05.2021-56733164
4. 11.01.2022-56733164
5. 19.06.2022-56733164/203
6. 20.12.2024-E-56733164-203-262963497
7. 20.12.2024-E-56733164-203-262963478
